# Outcome of Loupe-Assisted Sub-inguinal Varicocelectomy in Infertile Men

**DOI:** 10.5812/numonthly.7166

**Published:** 2012-12-15

**Authors:** Ju Seo

**Affiliations:** 1 Department of Urology, Cheil General Hospital, Kwandong University College of Mediine, Seoul, South Korea

**Keywords:** Varicocele, Infertility

Dear Editor,

In report on varicocele and infertility (1, 2), varicocele treatment should be offered to the male partner of a couple attempting to conceive, when all of the following are present: 1) a varicocele is palpable; 2) the couple has documented infertility; 3) the female has normal fertility or potentially correctable infertility; and 4) the male partner has one or more abnormal semen parameters or sperm function test results. If a varicocele is palpable in physical examination, color doppler may not be necessary.

Most experts perform inguinal or subinguinal microscopic varicocelectomy. Techniques using optical magnification maximize preservation of arterial and lymphatic vessels while reducing the risk of persistence or recurrence of varicocele (3). Varicocelectomy is associated a small risk of wound infection, hydrocele, persistence or recurrence of varicocele and, rarely, testicular atrophy.

Microscopic varicocelectomy successfully eliminates over 90% of varicocele. Most studies have reported that semen quality improves in a majority of patients following varicocele repair. Surgical correction of varicocele results in significant improvement in semen parameters in up to two thirds of afflicted men, and 30% to 60% of the couples will achieve pregnancy as a result of varicocelectomy. If clinical center had not microscopic instrument, Loupe assisted varicocelectomy might be useful.
